# The new metrics and additional objectives of the Jornal Brasileiro de Pneumologia

**DOI:** 10.36416/1806-3756/e20240284

**Published:** 2024-09-16

**Authors:** Marcia Pizzichini, Bruno Guedes Baldi

**Affiliations:** 1. Programa de Pós-Graduação em Ciências Médicas, Universidade Federal de Santa Catarina, Florianópolis (SC) Brasil.; 2. Divisão de Pneumologia, Instituto do Coração - InCor - Hospital das Clínicas, Faculdade de Medicina, Universidade de São Paulo, São Paulo (SP) Brasil.

The *Jornal Brasileiro de Pneumologia* (JBP) is about to celebrate 50 years of existence. However, it was only in the last two decades that the JBP embarked on a consistent trajectory towards increasing international recognition. This was possible because of the enormous effort of investigators, academicians, and public health professionals who engaged with editors, associated editors, and reviewers in a journey to increase the excellence of the dissemination of knowledge on respiratory medicine and correlated areas in the JBP.

The recent publication of indicators for the JBP by the two major international indexing agencies--Scimago Journal & Country Rank (SJR) and Journal Citation Reports (JCR) Web of Knowledge, Clarivate Analytics-supports this statement and is a source of pleasure for our scientific community because the JBP have continued attaining higher levels when compared with previous years.^1^ ^,^ ^2^ Therefore, these results certainly expand our attention for the submission of manuscripts by national and international authors, who may contribute to increase the quality of our Journal.

According to the 2023 SJR, the JBP ranked 18th among 443 scientific Brazilian journals within all subject areas. In 2022, the JBP ranked 47th. Additionally, according to the JCR, a database that includes international pulmonary medicine journals from different countries, the two-year citation index of the JBP increased from 2.7 in 2022 to 2.9 in 2023. It is important to reinforce that the trend for many journals was to have a decrease in the impact factor that coincided with the end of the COVID-19 pandemics. Consequently, the JBP ranking in the JCR rose from 47th to 39th among 107 international journals in the area of respiratory medicine, and from 14th to 9th among the 391 Brazilian journals in all subject areas. Given these newly issued indicators, we expect that the JBP will move up to category A4 in Medicine I in the journal ranking system of the Brazilian Office for the Advancement of Higher Education, a system known as Qualis.^3^

Another aspect worth mentioning is that although most manuscripts submitted to the JBP are from Brazil (70%), we have increasingly been receiving submissions from countries of all continents. Despite international visibility is always in the JBP editors’ mind, we will continue to encourage submissions and publications of translational pulmonary and respiratory medicine that have novel, ethical, and strong methodology from local and international researchers. Additionally, the JBP aims to publish other articles that have clinical applicability for pulmonologists and specialists in related areas, obviously maintaining scientific rigor.

The JBP is the only Latin American journal in the pulmonary and respiratory medicine field; its continuous publication and the access to all of the articles is free of charge. To be fully free of charge, the JBP is financially supported by the *Sociedade Brasileira de Pneumologia e Tisiologia* (SBPT, Brazilian Thoracic Society), the largest Latin American Respiratory Society, comprising about 4,000 associates and, occasionally, by Governmental Funding Agencies such as the *Brazilian Conselho Nacional de Desenvolvimento Científico e Tecnológico* (CNPq, National Council for Scientific and Technological Development).^4^ However, these very agencies have budget constraints that limit the growing capacity of Brazilian researchers. In synthesis, we urgently need improvements to support meaningful research across the inequalities of our country to increase the quality of our publications even more.

There are also other invisible aspects of the JBP that are related to the quality of publications and, consequently, to the impact factor. The board of editors has national and international researchers, the reviewers of the articles submitted are blinded regarding authorship, and the JBP follows the WHO and the International Committee of Medical Journal Editors (ICMJE) policies in regard to authorship, registrations of clinical trials, use of artificial intelligence in research, knowledge dissemination, ethical principles, and rules for publication transparency.^5^

Finally, a recent trend for and a future direction of the JBP has been the expansion of its educational arm to graduate students through appraisal of selected papers and invitation of young academic researchers to become associated reviewers. We also seek to continue debating topics on issues of importance in pulmonary and correlated areas that are relevant for public health policies in underdeveloped countries. Other recent achievements are the publication of manuscripts only in English, the inclusion of a series related to pediatric respiratory care, the increase in social media dissemination of knowledge with periodic podcasts about relevant publications, and the effort to increase the number of guidelines and consensuses on relevant issues published according to priorities established in accordance with the SBPT agenda. The focus and direction of the JBP are to expand our international status of excellence in respiratory and pulmonary medicine and, in a certain way, to meet the needs of the SBPT planning and respiratory researchers. In addition, we hope to help the practice of professionals related to the respiratory area whenever possible.
